# Outpatient treatment of acute poisonings in Oslo: poisoning pattern, factors associated with hospitalization, and mortality

**DOI:** 10.1186/1757-7241-20-1

**Published:** 2012-01-04

**Authors:** Cathrine Lund, Odd M Vallersnes, Dag Jacobsen, Oivind Ekeberg, Knut E Hovda

**Affiliations:** 1 Department of Acute Medicine, Oslo University Hospital Ullevaal, Kirkeveien 166, Oslo, (0407), Norway; 2 Emergency Medical Agency, City of Oslo, Municipality of Oslo, Storgata 40, Oslo, (0182), Norway; 3 General Practice Research Unit, University of Oslo, Oslo, P.O. Box 1072 Blindern, Oslo, (0316), Norway; 4 National Center for NBC Medicine, Department of Acute Medicine, Oslo University Hospital Ullevaal, Kirkeveien 166, Oslo, (0407), Norway

**Keywords:** Emergency Medical Services, Emergency Treatment, Outpatient Clinics, Outpatients, Street Drugs, Toxicology, Triage

## Abstract

**Background:**

Most patients with acute poisoning are treated as outpatients worldwide. In Oslo, these patients are treated in a physician-led outpatient clinic with limited diagnostic and treatment resources, which reduces both the costs and emergency department overcrowding. We describe the poisoning patterns, treatment, mortality, factors associated with hospitalization and follow-up at this Emergency Medical Agency (EMA, "Oslo Legevakt"), and we evaluate the safety of this current practice.

**Methods:**

All acute poisonings in adults (> or = 16 years) treated at the EMA during one year (April 2008 to April 2009) were included consecutively in an observational study design. The treating physicians completed a standardized form comprising information needed to address the study's aims. Multivariate logistic regression analysis was used to identify the factors associated with hospitalization.

**Results:**

There were 2348 contacts for 1856 individuals; 1157 (62%) were male, and the median age was 34 years. The most frequent main toxic agents were ethanol (43%), opioids (22%) and CO or fire smoke (10%). The physicians classified 73% as accidental overdoses with substances of abuse taken for recreational purposes, 15% as other accidents (self-inflicted or other) and 11% as suicide attempts. Most (91%) patients were treated with observation only. The median observation time until discharge was 3.8 hours. No patient developed sequelae or died at the EMA. Seventeen per cent were hospitalized. Gamma-hydroxybutyric acid, respiratory depression, paracetamol, reduced consciousness and suicidal intention were factors associated with hospitalization. Forty-eight per cent were discharged without referral to follow-up. The one-month mortality was 0.6%. Of the nine deaths, five were by new accidental overdose with substances of abuse.

**Conclusions:**

More than twice as many patients were treated at the EMA compared with all hospitals in Oslo. Despite more than a doubling of the annual number of poisoned patients treated at the EMA since 2003, there was no mortality or sequelae, indicating that the current practice is safe. Thus, most low- to intermediate-acuity poisonings can be treated safely without the need to access hospital resources. Although the short-term mortality was low, more follow-up of patients with substance abuse should be encouraged.

## Background

Efficiency and cost control are both universal political health goals. In Norway, one approach is to treat patients at the lowest health care level possible without impairing the quality of the treatment. Recognizing and hospitalizing high-risk patients are crucial to an effective system.

Acute poisoning is considered a major health problem worldwide and is a frequent cause of hospital admission. Most patients are managed as outpatients in hospital emergency departments. In Scandinavia, however, patients presenting with poisoning by substances of abuse, especially heroin overdose, are frequently discharged by the ambulance service without transfer to hospital. Oslo has a long tradition of treating acute poisoning by substances of abuse in a unique outpatient clinic, the Emergency Medical Agency (EMA, "Oslo Legevakt"). This physician-led walk-in clinic has served the entire city 24 hours a day, seven days a week since 1900 and treats low- to intermediate-acuity patients who would otherwise present to hospital emergency departments. Although similar in many ways to an emergency department, the EMA is not hospital based. Because the diagnostic tools and treatment options are limited, treatment at the EMA is less resource consuming than in-hospital treatment of equivalent conditions.

Ambulance paramedics triage patients to hospital emergency departments or the EMA based on the patient's clinical condition. Low-acuity patients presenting directly to hospital emergency departments are often referred to the EMA for initial evaluation. Patients are often assessed at the EMA before hospital admission, giving the clinic a gatekeeping function for hospital emergency departments. We are not aware of similar clinics, and a comprehensive literature search did not reveal reports of equivalent treatment units.

The EMA provides treatment for poisoning by substances of abuse, and the poisoning pattern here reflects the most frequent and dangerous substances of abuse presently in use [1]. In 2003, the EMA treated the same number of acute poisonings as all Oslo Hospitals in total, the mortality was zero and most patients were discharged after a short observation period [2].

Although the low-level care at the EMA is efficient, it is important to avoid adversely affecting patient safety. Evaluating the mortality at the EMA and after discharge may have implications in terms of deciding whether to discontinue or expand the current practice. If the low-level care provided at the EMA is safe, this may identify ways for more efficient handling of these patients in emergency departments. Because poisoning patterns change with time, updated information about poisoning trends is important for clinicians and for establishing preventive initiatives. The mortality rates 10 and 20 years after acute poisoning are alarmingly high, reflecting the importance of adequate follow-up initiatives [3,4]. Referral to follow-up after treatment at the EMA has not been studied, and it is unknown whether the current practice is adequate.

The study objectives were (1) to describe all acutely poisoned adults presenting at the EMA in one year beginning in April 2008 by collecting data on the number of patients, toxic agents, evaluated intention and complications; (2) to evaluate the treatment given and to identify factors associated with hospitalization and referral to follow-up; and (3) to discuss whether treatment of acute poisonings at the EMA may be considered safe with respect to mortality at the EMA and after one month.

## Methods

### Study Design

This study was part of a larger observational cross-sectional multicentre study conducted from 15 April 2008 to 14 April 2009 at the EMA, the five hospitals in Oslo that treat poisoned patients and the Institute of Forensic Medicine. The aim was to obtain an updated complete epidemiological picture of acute poisonings in the City of Oslo, the Municipality of Oslo, Norway. This paper presents data from the EMA. Data on the hospitalized patients (n = 1069) will be published separately (Lund C, Teige B, Drottning P, Stiksrud B, Rui TO, Lyngra M, Ekeberg O, Jacobsen D, Hovda KE: A one year observational study of all hospitalized and fatal acute poisonings in Oslo, submitted). The study was designed similar to a study conducted in 2003 with the intention of comparing results [2,5,6].

### Ethics

The study was approved by both the Norwegian Regional Ethics Committee and the National Data Inspectorate. Studying intoxicated patients is difficult from many perspectives, including acquiring written consent, because of the nature of the patients' behaviour and physical conditions. The study subjects were informed about the aims of the study and were given a written information leaflet with the name and phone number of the study coordinator. They were also given the right to refuse participation or to withdraw consent at any time without reprisal. Thereafter, their verbal consent was confirmed by the independent interviewing physician in a specific question included in the standardized interview form (described below).

### Setting

The study was performed at the EMA, an outpatient clinic serving the entire city 24 hours a day, seven days a week. It is located in central Oslo, 3.5 km from the nearest hospital. The clinic is similar to an emergency department, but has limited resources (e.g., gastric decontamination and intubation are not performed, and no blood gas analysis equipment is available). All patients are attended by physicians, and the observation limit is 24 hours. The physicians working at the EMA are general practitioners, mainly with a level of training comparable to hospital residents. In contrast to other outpatient walk-in clinics, the EMA also receives patients from ambulances. Ambulance paramedics triage patients to the EMA or hospital emergency departments. In cases of opioid overdoses, however, paramedics may administer naloxone on site without further transfer [2,7]. There are no standard triage criteria. This decision is based on an evaluation of the patient's clinical condition, knowledge of the toxic agents and the treatment options at the EMA. In general, stable patients likely to require observation for < 24 hours and likely not to require hospital treatment (e.g., with N-acetylcysteine, flumazenil, gastric decontamination, intubation or thorough laboratory testing) are brought to the EMA. Patients with symptoms and history of gamma-hydroxybutyric acid poisoning are usually hospitalized. Coma is a strong triage parameter for hospital admission unless it is caused by substances of abuse. The current principle is to treat patients at the lowest treatment level possible while still providing adequate care.

Oslo is the capital of Norway. The city area is 454 km^2^, and as of July 2008 the population was 568 809, of whom 466 423 were ≥ 16 years. The unemployment rate in Oslo was 2.2% in 2008 [8]. In terms of gross domestic product per capita, the World Bank ranked Norway as the fifth highest country in 2008 [9]. Drug and alcohol policies are strict in Norway. Alcohol is restricted and taxed heavily. Only the Wine and Spirits Monopoly may sell beverages exceeding 4.75% ethanol content, and off-license sales close at 8 pm on weekdays and 6 pm on Saturdays. Cannabis and other non-prescription substances of abuse are illegal. There is a methadone/buprenorphine maintenance programme for heavy users of opioids. The EMA is part of the National Public Health Care.

### Selection of Participants

All adults (≥ 16 years) presenting at the EMA with a primary diagnosis of acute poisoning were included consecutively. Because laboratory testing is not performed routinely at the EMA, inclusion was based on a clinical diagnosis of acute poisoning. Poisonings were defined as exposure to substances in assumed toxic amounts. Chronic poisonings were not included.

### Methods of Measurement, Data Collection and Processing

All physicians at the EMA participated in the data collection. All evaluations were made by the treating physician who completed a standardized form that included information to satisfy all of the study objectives. The study coordinator manually cross-checked the forms against the electronic patient list to ensure that all patients meeting the criteria were included. In the few cases where patients had either left the EMA or had been transferred to hospital before a form was completed, a form was completed based on the electronic medical journal. This journal was also used as a supplement where variables were missing. Missing variables were coded as unknown and excluded from that particular analysis. Some patients had two contacts on the same day for the same toxic agent. In these cases, only the first of the contacts was included.

The data were entered manually into an SPSS spreadsheet and checked systematically for errors. Ten per cent of the variables were cross-checked randomly with the original forms, and this cross-check showed 99.94% consistency.

### Outcome Measures

In patients exposed to more than one substance, the main toxic agent was defined as the substance suspected to be the most toxic in the amount assumed taken. This was the treating physician's clinical evaluation based on the information available including statements from the patient, companions, ambulance service, clinical observations or laboratory findings. Substances presumed to be less toxic were registered as co-agents. Carbon monoxide (CO) poisoning was classified as either CO/fire smoke or CO/engine smoke poisoning. Complications were defined according to standard clinical definitions similar to those used in the 2003 study [5]. Consciousness was measured on presentation and analysed as a categorical value. Coma was defined as < 8 on the Glasgow Coma Scale (GCS) and drowsiness as GCS 8-14.

The intention behind the act of poisoning was evaluated by the treating physician. Poisonings with substances of abuse, including heroin and ethanol for recreational purposes, were classified as accidental overdoses with substances of abuse (AOSAs). Suicidal motivation in the act of poisoning was classified as either a possible or definite suicide attempt. This distinction was based on whether the patient considered the toxic agent lethal and whether other measures had been taken to ensure a lethal outcome. If the motive was ambivalent (e.g., by seeking help shortly after ingestion), the poisoning was classified as a possible suicide attempt. No objective scales were used for this evaluation. Accidents included both external causes of poisoning and self-inflicted accidents (e.g., taking the wrong medication) where the agent had been taken for neither self-harm nor intoxication purposes.

Causes of death were obtained from the National Death Register. Fatalities within one month after the patient's last contact and after every contact were registered.

### Statistics

Pearson's chi-square or Fisher's exact test (cell values < 5) was used to compare frequencies. Age comparisons were made using the Mann-Whitney *U *test.

Univariate and multivariate logistic regression were used to identify clinical patient characteristics associated with hospital admission. Because we wanted to evaluate the physicians' decision making in treating the toxicological emergency, patients either transferred to a psychiatric ward or leaving against medical advice were excluded from this particular analysis. All variables included in the multivariate models were clinically relevant and had a p-value < 0.20 in the univariate analysis. The assumptions underlying multivariate logistic regression analyses were checked and found to be adequately fulfilled.

Univariate logistic regression was also used to analyse the association between the intentions behind the poisonings and the six major referral endpoints: hospitalization, transfer to a psychiatric ward, psychiatric outpatient treatment, other follow-up (including to a general practitioner, addiction clinic, emergency social services and other specialists), no referral and those leaving against medical advice. Only patients with unknown intention were excluded from this particular analysis.

In the calculation of short-term mortality, hospitalized patients were excluded because we wanted to calculate the mortality after treatment at the EMA. Hospitalized patients and those with missing social security number or permanent residence outside Norway were excluded; this reduced the numbers checked against the National Death Register to 1799/2348 contacts in 1410/1856 patients. Findings with p-values < 0.05 were considered significant. SPSS 16.0 (SPSS Inc., Chicago, IL, USA) was used to analyse the data.

## Results

Of 2401 adults presenting at the EMA with an acute poisoning, 53 (2.2%) refused participation. These patients did not differ significantly from the studied population in terms of age, sex, intention and main toxic agents. Of the 17 patients with two contacts on the same day because of the same toxic agent, five had left against medical advice during the first contact, and four of the second contacts had resulted in a higher level of follow-up.

**Figure 1:**
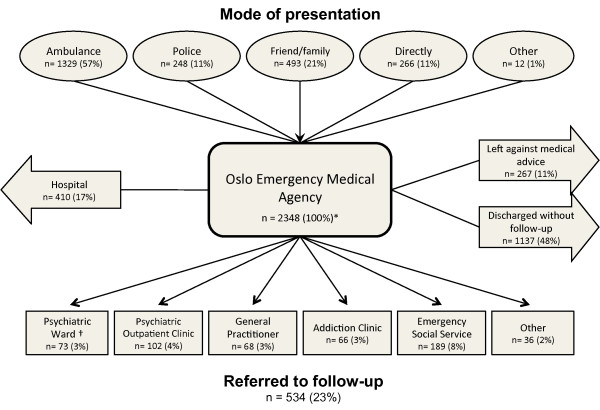
**Patient flow in 2348 cases of acute poisoning presenting at the EMA during one year**. *Excludes 53 subjects who refused to participate †29 transferred under paragraph of compulsory observation.

There were 2348 poisoning episodes in 1856 individuals (Table 1). Of these individuals, 1157 (62%) were male. The median age was 34 years (range 16-96) overall: 37 years for males and 29 years for females (p < 0.001). Figure 1 illustrates the patient flow at the EMA. The patients hospitalized from the EMA comprised 37% of all hospitalized acute poisonings in Oslo (n = 1069) (Lund C, Teige B, Drottning P, Stiksrud B, Rui TO, Lyngra M, Ekeberg O, Jacobsen D, Hovda KE: A one year observational study of all hospitalized and fatal acute poisonings in Oslo, submitted).

**Table 1:** Demographic data

	Males n (%)	Females n (%)	p-value	Total n (%)
**Known ID**	1125 (97)	694 (99)	0.002*	1819 (98)
Unknown ID	32 (3)	5 (1)	0.002*	37 (2)

**Episodes:**				
1	958 (83)	635 (91)	< 0.001*	1593 (86)
2-5	152 (13)	56 (8)	< 0.001*	208 (11)
6-22	15 (1)	3 (< 0.5)	ns	18 (1)

**Age:**				
16-25	274 (24)	311 (44)	< 0.001*	585 (32)
26-35	303 (26)	152 (22)	0.035*	455 (25)
36-45	243 (21)	102 (15)	< 0.001*	345 (19)
46-55	173 (15)	69 (10)	0.002*	242 (13)
56-65	89 (8)	33 (5)	0.014*	122 (7)
66-75	33 (3)	15 (2)	ns	48 (3)
> 75	15 (1)	13 (2)	ns	28 (2)
Unknown	27 (2)	4 (1)	0.004*	31 (2)

**Residency:**				
Oslo	757 (65)	528 (76)	< 0.001*	1285 (69)
Outside Oslo	328 (28)	158 (23)	0.007*	486 (26)
Homeless	20 (2)	2 (0)	0.006*	22 (1)
Outside Norway	22 (2)	6 (1)	ns	28 (2)
Unknown	30 (3)	5 (1)	0.004*	35 (2)

**Total individuals**	1157 (100)	699 (100)		1856 (100)

* Significant

ID: identification

The most frequent main toxic agents were ethanol (n = 1018; 43%), opioids (n = 519; 22%) and CO/fire smoke (n = 231; 10%) (Table 2). More than one agent was taken by 726 patients (31%), and the most common combination was benzodiazepines and opioids (n = 180; 8%). The most common co-agents were benzodiazepines (n = 254; 11%), ethanol (n = 218; 9%) and amphetamines (n = 123; 5%).

**Table 2:** Main and co-agents, main agents separated by sex

Toxic agent	Main+co-agents	Main agents			
	**n (%)**	**Total n (%)**	**Males n (%)**	**Females n (%)**	**p-value**

Ethanol	1236 (53)	1018 (43)	688 (44)	330 (42)	ns
Opioids	613 (26)	519 (22)	415 (27)	104 (13)	< 0.001*
CO/fire smoke	233 (10)	231 (10)	136 (9)	95 (12)	0.018*
Benzodiazepines	376 (16)	122 (5)	72 (5)	50 (6)	ns
Amphetamines	196 (8)	73 (3)	63 (4)	10 (1)	< 0.001*
Paracetamol	104 (4)	58 (2)	17 (1)	41 (5)	< 0.001*
GHB	58 (2)	54 (2)	39 (3)	15 (2)	ns
Antidepressants	54 (2)	39 (2)	10 (1)	29 (4)	< 0.001*
Scopolamine	45 (2)	34 (1)	26 (2)	8 (1)	ns
Neuroleptics	42 (2)	29 (1)	7 (< 0.5)	22 (3)	< 0.001*
Zopiclone/zolpidem	65 (3)	27 (1)	11 (1)	16 (2)	0.011*
Cocaine	32 (1)	12 (1)	10 (1)	2 (< 0.5)	ns
NSAIDs	31 (1)	12 (1)	6 (< 0.5)	6 (1)	ns
Antihistamines	24 (1)	10 (< 0.5)	2 (< 0.5)	8 (1)	0.004*
Antiepileptics	16 (1)	9 (< 0.5)	3 (< 0.5)	6 (1)	ns
Ecstasy	14 (1)	5 (< 0.5)	3 (< 0.5)	2 (< 0.5)	ns
Other gases	21 (1)	20 (1)	12 (1)	8 (1)	ns
Other	194 (8)	76 (3)	33 (2)	43 (5)	< 0.001*

**Total cases**		2348 (100)	1553 (100)	795 (100)	

*Significant

Main agents present in ≤ 5 patients were defined as "other"

NSAIDs: non-steroidal anti-inflammatory drugs

Among the opioids as main agent, 478 were by illegal substances. Among the legal opioids, 19 were by methadone and 14 were by buprenorphine. Among the cases involving antidepressants as the main agent, 22 were by selective serotonin reuptake inhibitors and five were by tricyclic antidepressants. Among the cases involving paracetamol as the main agent, 21 were by codeine-combination formulas

AOSAs accounted for 1714 (73%) contacts and was the major intention category in both men and women; 346 (15%) were accidental poisonings and 270 (11%) had a possible or definite suicidal intention behind the poisoning (Table 3). AOSAs were more common among men, whereas suicide attempt was more common among women. Age did not differ significantly between the intention categories.

**Table 3:** Physician's evaluation of intention

Intention	Males n (%)	Females n (%)	p-value	Total n (%)
AOSA	1259 (81)	455 (57)	< 0.001*	1714 (73)
Accident	194 (12)	152 (19)	< 0.001*	346 (15)
Possible suicide attempt	43 (3)	105 (13)	< 0.001*	148 (6)
Definite suicide attempt	46 (3)	76 (10)	< 0.001*	122 (5)
Unknown	11 (1)	7 (1)	ns	18 (1)

**Total cases**	1553 (100)	795 (100)		2348 (100)

*Significant

AOSA: accidental overdose with substances of abuse

On presentation, 1161 (49%) were awake, 1066 (45%) were drowsy and 120 (5%) were comatose. Complications developed in 341 (15%) cases. Of these, the most common were hypothermia (n = 104; 30%), respiratory depression (n = 71; 21%), hallucinations (n = 57; 17%), hypotension (n = 34; 10%), hypoglycaemia (n = 21; 6%) and seizures (n = 19; 6%). No patient had any observed sequelae. Complications were most frequent in patients with scopolamine (n = 26/34), cocaine (n = 5/12) or ecstasy (n = 2/5) as the main toxic agent.

Most patients received no further treatment beyond observation (n = 2133; 91%). Antidote(s) were given to 203 (9%), activated charcoal to 45 (2%), intravenous fluids to 18 (1%), anti-emetics/analgesics to nine (0.4%) and non-invasive mask ventilation to seven (0.3%) patients. Intubation and gastric decontamination are not performed at the EMA. One patient required cardiopulmonary resuscitation and naloxone in the ambulance but was awake upon arrival at the EMA and left against medical advice. He was alive one month later. Of the patients hospitalized from the EMA, 72 (18%) received treatment before hospital admission.

Naloxone was given by the ambulance service or at the EMA intramuscularly to 123 (5%), intravenously to 16 (1%) and by both routes to 63 (3%) patients. Five (0.2%) received flumazenil. No other antidotes were administered. One patient with scopolamine-induced anti-cholinergic syndrome misdiagnosed as acute drug-induced psychosis developed hypotension and coma after treatment with levomepromazine. There were no other treatment-related complications. The median observation period was 3.8 hours (inter quartile range 2.1-4.7) for discharged patients.

Four hundred and ten (17%) of the patients assessed at the EMA were hospitalized (Figure 1). Factors associated with hospital admission were GHB or paracetamol as the main toxic agent, a possible or definite suicidal intention and complications, especially respiratory depression and reduced consciousness (Table 4). Ethanol and opiates were associated with discharge.

**Table 4:** Factors associated with hospitalization, results of multivariate logistic regression

			Crude		Adjusted	
	**Total**	**Hospitalized**	**OR**	**95% CI**	**OR**	**95% CI**

**Age **(+10 years)			1.0	0.9-1.0	1.1**	1.0-1.2

**Sex**						
Males	1319	237 (18)	Ref			
Females	689	173 (25)	1.5*	1.2-1.9	1.1	0.8-1.5

**Main agents**						
Other agents	609	188 (31)	Ref			
Ethanol	859	65 (8)	0.2*	0.1-0.3	0.2*	0.1-0.3
Opioids	437	76 (17)	0.5*	0.3-0.7	0.4*	0.3-0.6
GHB	48	40 (83)	11.2*	5.1-24.4	12.5*	5.4-29.3
Paracetamol	55	41 (75)	6.6*	3.5-12.3	3.8*	1.9-7.6

**GCS**†						
Awake (15)	990	206 (21)	Ref			
Drowsy (8-14)	908	167 (18)	0.9	0.7-1.1	1.6**	1.2-2.3
Comatose (< 8)	109	37 (34)	2.0**	1.3-3.0	2.3**	1.3-4.3

**Complications**						
Number	1726	299 (17)	Ref			
Respiratory depression	61	33 (54)	5.6*	3.3-9.5	7.8*	4.2-14.4
Others	221	78 (35)	2.6*	1.9-3.5	3.7*	2.5-5.3

**Intention**						
Accidents	334	70 (21)	Ref			
AOSA	1439	200 (14)	0.6**	0.5-0.8	0.9	0.6-1.4
Possible suicide attempt	133	70 (53)	4.1*	2.7-6.4	4.5*	2.8-7.3
Definite suicide attempt	88	59 (67)	7.7*	4.6-12.9	8.2*	4.7-14.4
Unknown	14	11 (79)	13.8*	3.8-50.9	2.8	0.5-17.2

**Total cases**	2008	410 (20)				

*Significant, p < 0.001, **significant, p < 0.05

AOSA: accidental overdose with substances of abuse

†Glasgow Coma Scale: one case with a missing value

Age was subtracted by 10 in the analysis

Forty-eight per cent (n = 1137) were discharged from the EMA without plans for follow-up, and 11% (n = 267) left against medical advice (Figure 1, Table 5). Suicide attempters were most likely to be either hospitalized (n = 129; 48%) or referred to psychiatric follow-up (n = 100; 37%). Patients with an AOSA or other accidental poisoning were more likely to be discharged without follow-up compared with suicide attempters: OR 22.6 (CI 12.8-39.8) and OR 29.8 (CI 16.4-54.2). Patients with an AOSA were also more likely to receive non-psychiatric follow-up or to leave against medical advice: OR 1.9 (CI 1.3-3.0) and OR 9.0 (CI 3.7-21.9), respectively.

**Table 5:** Odds for different follow-up according to intention; results of univariate logistic regression

Intention	Hospitalized	Psychiatric Ward	Psychiatric Outpatient Clinic	Other	No referral	Left
**Suicide attempt**	48%	16%	21%	9%	5%†	2%
Reference						

**AOSA**	12%	2%	2%	17%	53%	15%
OR (95% CI)	0.14* (0.1-0.2)	0.08* (0.1-0.1)	0.08* (0.1-0.1)	1.94** (1.3-3.0)	22.59* (12.8-39.8)	8.97* (3.7-21.9)

**Accident**	20%	< 0.5%	3%	13%	60%	3%
OR (95% CI)	0.28* (0.2-0.4)	0.02* (0.0-0.1)	0.11* (0.1-0.2)	1.44 (0.9-2.4)	29.80* (16.4-54.2)	1.74 (0.6-5.1)

**Total**	17%	3%	4%	15%	48%	11%

*Significant, p < 0.001, **significant, p < 0.05

AOSA: accidental overdose with substances of abuse

Eighteen patients lacked recorded intention and were excluded from the analysis. More than one follow-up is possible for each patient

†One patient with a definite suicide attempt

No patient died at the EMA. Nine patients had fatal outcomes within one month after their last episode, giving a one-month mortality rate of 0.6%. Because of repetition in three patients, 14 contacts had fatal outcomes within one month, resulting in a one-month relative mortality rate of 0.8% per episode. These three had been admitted previously for uncomplicated poisonings with ethanol, benzodiazepines and opioids, and had been discharged without follow-up. The nine patients had a median age of 31 years (range 25-69) and six (67%) were male (ns). Five patients died during the first three days. Of the nine patients, five died of new overdoses (> 2 days after discharge), two died of unrelated causes, one committed suicide and one may have died of opioid toxicity recurrence after treatment at the EMA. This latter patient was found dead in his apartment a few hours after discharge after treatment with 0.8 mg naloxone both intramuscularly and intravenously for an AOSA with heroin (statement from the patient and clinical signs). He had been observed for four hours, and a referral letter had been sent to a psychiatric outpatient clinic. At the autopsy, the cause of death was found to be methadone poisoning. It is uncertain whether this death was caused by a new overdose with methadone or if it initially was an overdose with methadone misinterpreted as heroin. The patient who committed suicide had been discharged from the EMA 10 days earlier after a possible suicide attempt with ethanol and had been referred to a psychiatric outpatient clinic. There is no information about whether this patient had received an appointment. Mortality did not differ significantly between patients referred to follow-up (5/391; 1.3%) and those discharged without referral (3/850; 0.4%, p = 0.12) or those who left against medical advice (1/169; 0.6%, p = 1.00).

## Discussion

The EMA treated more than twice the number of patients compared with all hospitals in Oslo. Accidental poisonings with substances of abuse predominated. One of five patients required hospitalization. The factors associated with hospitalization were GHB or paracetamol as the main toxic agent, suicidal intention, presence of complications and reduced consciousness. Almost half of the patients were discharged without plans for follow-up. None died at the EMA, and the one-month mortality was 0.6%.

The large proportion of acute poisoning treated at the EMA compared with Oslo hospitals is a new and interesting finding. The number of acute poisonings treated at the EMA in 2008 has more than doubled (146% increase) since 2003 (956 in 2003 vs. 2348 in 2008) [2]. In the same period, the number of acute poisonings treated in Oslo hospitals increased by about 10% (947 in 2003 vs. 1069 in 2008) (Lund C, Teige B, Drottning P, Stiksrud B, Rui TO, Lyngra M, Ekeberg O, Jacobsen D, Hovda KE: A one year observational study of all hospitalized and fatal acute poisonings in Oslo, submitted). Although the ambulance management denies a shift in triage pattern, a lower threshold for ambulance transfer to the EMA is a possible explanation for the increase. If so, the percentage of patients hospitalized from the EMA would be expected to have decreased from 2003 to 2008, which was not the case (16% and 17%, respectively). The proportion of patients arriving at the EMA by ambulance increased by 38%. However, this cannot explain the 146% increase in poisonings of patients treated at the EMA. Although other factors, such as increased availability of illegal drugs, may have increased somewhat, this would have contributed only to a minor extent.

Although prescription drugs comprised most poisonings in patients treated at the Oslo hospitals, substances of abuse predominated at the EMA. The main agents were largely unchanged compared with 2003 apart from an increase in CO/fire smoke poisonings, which may be explained by 23% more building/house fires in 2008 [10]. The present male predominance, unchanged from 2003, reflects the higher prevalence of substance abuse among males [2,11]. In contrast to hospital-treated poisonings, which comprise mainly suicide attempts [6,12-14], AOSAs predominated at the EMA.

The complication rate was lower in the EMA than in the Oslo hospitals (18%) and was unchanged from 2003 [2,5]. Combined with the lack of deaths at the EMA, this finding shows that critically ill patients were hospitalized directly, in accordance with the intended patient flow. The low percentage of comatose patients (13% in 2003 vs. 5% in 2008, p < 0.001) [2] might relate to either a lower threshold for ambulance transfer to the EMA or the observed increase in antidote administration on site by the ambulance service. Interestingly, a larger percentage received naloxone (6% in 2003 vs. 9% in 2008, p = 0.004), although the percentage of poisonings by opioids remained unchanged.

The factors associated with hospitalization reflect the policy at the EMA to treat efficiently a large number of uncomplicated conditions. N-acetylcysteine administration and intubation are restricted to hospital treatment because of potential complications. Opioids were associated with discharge (83%). A comparison with ambulance data showed large variation in hospitalization practices for treating opioid poisonings, from 9229/11336 (81%) refusing transport in an Australian study [15], to 295/1087 (27%) discharged in an Austrian study [16]. This variability may reflect a more liberal tradition to either accept the patient's wish to be discharged or to consider further treatment unnecessary.

One positive finding was that few patients with a suicidal intention were discharged without follow-up; this is important because a previous suicide attempt is the strongest predictor of completing suicide [17,18]. However, considering the high repetition and mortality rates in this particular group [3,4,19], it is disturbing that half of the patients with an AOSA were discharged without follow-up. A similar low percentage of patient referral was reported both after hospital treatment of AOSAs in Oslo (63%) and in a Swiss study of opiate addicts treated for acute overdose (33%) [20,21]. In most cases, the treating physician had deemed immediate follow-up unnecessary, although in many cases, no suitable options were available. Although motivation may be a challenge, there are definite follow-up alternatives in Oslo for overdose victims without suicidal intent. The most relevant are outpatient units for substance abusers, social security services or visits to general practitioners who have the main responsibility for coordinating treatment procedures.

Although no patients died at the EMA, one may have died from methadone toxicity recurrence after discharge. The lack of laboratory data makes it impossible to determine whether methadone or heroin was the initial compound. Because of the long-acting effect of methadone (36-48 hours), fatal outcomes have been reported in hospital several hours after discontinuation of naloxone infusion thought to be sufficient [22]. The duration of naloxone treatment varies between 45 minutes and four hours, depending on the dose and route of administration [23]. Our patient had been observed for four hours without any symptoms. However, naloxone had been given both intravenously and intramuscularly, which may have delayed the onset of symptoms. It is uncertain whether a laboratory test in an emergency department would have made a difference. A challenge with many patients is that they wish to leave during observation because of abstinence and cannot be held back unless they are actively suicidal, psychotic or clearly in a life-threatening condition. In 2003, none of the poisonings treated at the EMA was fatal [2].

The one-month mortality was highest in the first three days following discharge, and two-thirds of the deaths were caused by new overdoses, suggesting a target for preventive initiatives. The mortality did not differ significantly between patients referred to follow-up and those not referred, indicating the difficulty in assessing short-term mortality risk. On the other hand, the study may indicate that physicians are more concerned about suicide risk than the risk of mortality associated with substance abuse. However, we do not know how long patients have to wait for their appointments or whether they show up. Moreover, data on the effects of different follow-up treatments on mortality are scarce.

It is debated whether treatment in an outpatient clinic is preferable to treatment in an emergency department. Access to a wide range of resources is practical but may lead to overuse. Laboratory results have limited value in the clinical setting [24]. Patients brought first to the EMA and thereafter hospitalized in an ambulance may present an extra burden on transport resources. The vast majority, however, were discharged from the EMA after a short observation period. According to a World Health Organization report, the advantages of specialist treatment are not observed for outpatient care [25]. This report concludes that health care systems with a strong primary care orientation use services more appropriately, at lower cost and improve health outcomes; however, the report also emphasizes the limited evidence about what can be shifted from specialist-led secondary care to primary care. In addition, high volumes of low-acuity patients in emergency departments may compromise the ability to provide high-quality emergency care to high-acuity patients because of overcrowding [26]. A separate outpatient clinic may reduce waiting times and the number of low-acuity patients leaving without being seen.

We found no evidence to support the concept that the limited resources at the EMA may have impaired treatment quality. A low complication rate and low (possibly zero) mortality indicate that the current practice is safe and show how low- to intermediate-acuity poisonings can be treated effectively using limited diagnostic and treatment resources. However, preventing repetition is an important aspect that needs to be addressed, and further studies should focus on whether patients complete their designated follow-up and on the effect of follow-up treatment. Although local health care structures and poisoning epidemiology affect the generalizability of the study results, the observation procedure in use at the EMA may be applicable for practice in rural areas or as a template for more effective handling of these patients in hospital emergency departments.

This study has limitations. All physicians at the EMA assisted in patient inclusion. This was an advantage in ensuring complete inclusion but may have resulted in a higher interrater variability for variables such as evaluated intention. Despite the prospective inclusion, cross-checking of forms against electronic patient lists and thorough follow-up at the EMA, a few patients may have been missed. Because of the short observation time, complications and sequelae may have developed after discharge and hence may have been missed. An unknown social security number in 37 patients weakened the one-month mortality analysis. It is debatable whether both a verification of the toxic agents and an objective evaluation of intention should have been performed. The clinical practice at the EMA is not based on laboratory results or structured interviews, and we based this study on the parameters used in the actual clinical setting. Further, the limited value of laboratory results in the clinical setting has been shown [24].

## Conclusions

More than twice as many acute poisonings were managed at the EMA compared with all hospitals in Oslo, and the median stay was less than four hours. AOSAs in males predominated. The factors associated with hospitalization from the EMA were GHB or paracetamol as the main agent, a suicidal intention, respiratory depression and reduced consciousness. These factors reflect the current policy at the EMA to treat effectively a large number of uncomplicated conditions. Only half of the patients were offered follow-up appointments, and this is probably too low for a patient group with a high prevalence of substance abuse, suicidal behaviour and increased long-term mortality. The large proportion of patients treated but zero mortality and no sequelae indicate that the treatment at the EMA is safe. These data show that low- to intermediate-acuity patients can be treated effectively in a low-resource setting. A current ongoing study at the EMA focuses on whether patients continue with their designated follow-up and the effects of this follow-up treatment.

## Abbreviations

AOSA: accidental overdose with substances of abuse; CI: 95% confidence interval; EMA: the Emergency Medical Agency; GCS: Glasgow Coma Scale; GHB: gamma-hydroxybutyric acid; IQR: inter quartile range; OR: odds ratio.

## Competing interests

The authors declare that they have no competing interests.

## Authors' contributions

KEH, DJ and OE conceived the study and designed the trial. OMV supervised the conduct of the trial and data collection. CL managed and analysed the data, including quality control, and drafted the manuscript. All authors read and approved the final manuscript.
